# Sample size calculations for indirect standardization

**DOI:** 10.1186/s12874-023-01912-w

**Published:** 2023-04-11

**Authors:** Yifei Wang, Philip Chu

**Affiliations:** Department of Radiology, Department of Epidemiology and Biostatistics, University of California, San Francisco, San Francisco, USA

**Keywords:** Hospital profiling, Indirect standardization, Sample size calculation

## Abstract

Indirect standardization, and its associated parameter the standardized incidence ratio, is a commonly-used tool in hospital profiling for comparing the incidence of negative outcomes between an index hospital and a larger population of reference hospitals, while adjusting for confounding covariates. In statistical inference of the standardized incidence ratio, traditional methods often assume the covariate distribution of the index hospital to be known. This assumption severely compromises one’s ability to compute required sample sizes for high-powered indirect standardization, as in contexts where sample size calculation is desired, there are usually no means of knowing this distribution. This paper presents novel statistical methodology to perform sample size calculation for the standardized incidence ratio without knowing the covariate distribution of the index hospital and without collecting information from the index hospital to estimate this covariate distribution. We apply our methods to simulation studies and to real hospitals, to assess both its capabilities in a vacuum and in comparison to traditional assumptions of indirect standardization.

**Supplementary Information:**

The online version contains supplementary material available at 10.1186/s12874-023-01912-w.

## Introduction

Indirect standardization is an important tool for assessing the performance of a hospital (i.e., hospital profiling) compared to other hospitals in a wider population. This assessment is done by studying the incidence or prevalence of a (usually negative) binary outcome while adjusting for variables out of the hospital’s control which may confound comparison to other hospitals. For example, in the field of computed tomography (CT), there is currently a significant movement to standardize or optimize best practices between hospitals, especially with regards to radiation dosage, for safety quality assurance [1–4]. One of the most basic outcomes of interest in this movement is the incidence or prevalence of CT exams determined to be “high dose” in a hospital. Comparison of this number between hospitals, however, must take into account the part of the body being scanned and the size of the patient being scanned, both of which have: a) high impact on whether a high dose is acceptable and b) highly variable distributions from hospital to hospital.

Indirect standardization makes this comparison by studying the standardized incidence ratio (SIR), computed by dividing the observed incidence (or prevalence) of high dose exams in an index hospital by the expected incidence (or prevalence) of high dose exams if a wider population of reference hospitals shared the distribution of body part scanned and patient size seen in the index hospital [5]. The index hospital can then be considered “performing badly” if its SIR is substantially greater than 1, or equivalently if its observed incidence of “high dose” exams substantially out-populates the expected incidence of “high dose” exams. The utility of this methodology in hospital profiling is well-established [6–8].

Traditional methods of inference on this ratio view the denominator as fixed, modeling all uncertainty in its estimation as a consequence of uncertainty in the numerator [9–12]. The justifications for this assumption are numerous and multi-layered, but ultimately inadequate in a variety of circumstances [13–15]. They are especially inadequate when attempting to compute the sample size necessary to perform indirect standardization, as in such a context it’s usually the case that no data (or very little data) has thus far been observed from the hospital of interest.

The requirement of all or most of an index hospital’s data to perform indirect standardization can have severe consequences on how long it takes to profile a hospital or whether the profiling is done at all, as such a requirement not only presents logistical issues, but may also breach hospital policies on data-sharing and patient confidentiality [16]. The demand for an overall assessment of a hospital’s radiation dosage still persists, however, and the problem must be approached using novel methods.

In this paper, we explore the assumptions made when performing traditional inference on the SIR, explain how such assumptions can be inappropriate to our hospital profiling problem, especially in the context of sample size calculation, and present an alternative novel approach to SIR hypothesis testing that addresses the issues of traditional methods. We present a means of sample size calculation under this new approach, estimating how many exams are needed from the index hospital to consistently detect abnormally high rates of high dose exams.

Our sample size calculation methods are tested with an application using 157 example hospitals from which we have complete data, showing sample sizes computed using our method are sufficient but not excessive to achieve desired type I and type II error rates. We will also apply our methods to simulated hospitals, comparing the performance of our novel method to methods using assumptions associated with traditional indirect standardization.

## Methodology

We begin by describing our problem in the mathematical terms associated with traditional indirect standardization, then apply the language to our hospital profiling problem.

### Description of traditional indirect standardization and its short-comings

We assess the quality of a population of interest, the index, by studying a dichotomous outcome *Y*, controlled for a categorical predictive covariate *X*, which takes the values 1, ..., *J*. This categorical predictive covariate can denote a single variable, or a list of all combinations of the levels of multiple categorical variables. In the second case, the “distribution of *X*” is equivalent to the joint distribution of all constituent variables that form *X*.

Define the SIR as

1 $$\begin{aligned} \theta (\Lambda ,\textbf{p},q)=\frac{q}{\sum _{j=1}^J \lambda _j p_j} \end{aligned}$$

where

1. $\Lambda =\{\lambda _1,...,\lambda _J\}=\{\Pr (Y|X=1),...,\Pr (Y|X=J)\}$ in a large reference population. Information pertaining to this reference population can be taken from literature or can be estimated from large available databases (preferably, in the context of hospital profiling, the standard against which a hospital’s performance will be assessed)
2. $\textbf{p}=\{p_1,...,p_J\}=\{\Pr (X=1),...,\Pr (X=J)\}$ in the index population
3. $q=\Pr (Y)$ in the index population

Which constituencies of this ratio can be viewed as known exactly, and which must be viewed as uncertain estimates, depends on what we mean exactly by “population of interest”. We begin by describing interpretations in traditional indirect standardization [9–12].

Vector $\Lambda$ is viewed as known exactly, as indirect standardization assumes that the reference population has significantly greater sample size (or validity) than the index population, to a degree that any uncertainties in estimations made using information from the reference data (like $\Lambda$) are eclipsed by uncertainties in estimates using the index population.

Vector $\textbf{p}$ is also viewed as known exactly. Sometimes this viewpoint is motivated by the same high sample size assumptions made with $\Lambda$. Other times this viewpoint is a consequence of the “population of interest” being defined specifically by a collection of already-observed data points, as opposed to a population which has not been observed entirely, but from which we have sampled data. That is, using the language of hospital profiling, the “population of interest” would not be the index hospital itself, but a specific set of observed patients from said hospital. The estimated SIR, in such a case, would describe the quality of the index hospital’s care for the observed patients, rather than its overall quality of care. Under such traditional assumptions, the distribution of *X* naturally does not need to be estimated.

Value *q* is viewed as unknown. Even under cases where $\textbf{p}$ is known, the purpose of the SIR is to describe the underlying mechanisms that the population of interest uses to achieve its outcome prevalence. Such mechanisms may not be deterministic, even when $\textbf{p}$ is known. In the context of our hospital profiling problem, this refers to the fact that radiation dosage is highly variable even when physically identical patients are scanned at the same hospital in the same anatomic area, due to a combination of inconsistencies in execution of radiological protocols and the intrinsic randomness of radiation dosage.

Our hospital profiling problem can mostly follow the same standards on which components of the SIR to view as known and unknown, excepting the case of $\textbf{p}$, which we cannot view as being known exactly. In the context of sample size calculation, the reason for this is clear - we’ve never observed any data from the index hospital. Given sufficiently generous resources, it may be possible to pursue some preliminary study on $\textbf{p}$ to construct some anticipation of its true value. In fact, if the goal were only to construct a confidence interval of the SIR after collection of data, the denominator of the SIR may be estimated (with uncertainty) using the same collected data meant to estimate *q*, and literature exists to quantify said uncertainty in various respects [13–15]. However, even when such preliminary studies for sample size calculation are logistically possible (which itself is unlikely), they are unlikely to acquire the covariate distribution of the entire index hospital, leaving us with an estimate of $\textbf{p}$ that uses a sample of the index hospital’s patients, even though the population of interest is the entire index hospital. This problem usually persists even after data collection for the“main” analysis, as due to a variety of logistical and legal issues [16], it is possible that the collected data would not be a census of all exams performed at the index hospital.

### Proposed solution

Our hospital profiling problem seeks to compute the sample size necessary to detect hospitals with substantially more cases of high-dose exams than expected - that is, we seek to detect hospitals with SIR substantially higher than 1. This will be done using two mathematical statements, the proofs of which may be found in the appendix. All notation in this section are identical to those described in Description of traditional indirect standardization and its short-comings section.

#### Lemma 1

In an arbitrary index hospital, let $\theta (\Lambda ,\textbf{p},q)$ denote its SIR with respect to a reference population. Let $\hat{q}$ and $\hat{{\textbf {p}}}=\{\hat{p}_1,...,\hat{p}_J\}$ respectively denote estimated values for *q* and $\textbf{p}=\{p_1,...,p_J\}$, computed using observed prevalences of the outcome and each category of the predictive covariate, respectively, using a sample of *n* individuals from the index hospital.

The estimator $\hat{\theta }(\Lambda ,\hat{{\textbf {p}}},\hat{q})$ for $\theta$ has the following asymptotic distribution

2 $$\begin{aligned} \sqrt{n}(\hat{\theta }(\Lambda ,\hat{{\textbf {p}}},\hat{q}) -\theta (\Lambda ,\textbf{p},q)) \rightarrow N(0,\nabla \theta ^T\Sigma \nabla \theta ) \end{aligned}$$

where

3 $$\begin{aligned} \Sigma = \left[ \begin{array}{cc} q(1-q) &{} 0 \\ 0 &{} D_{\textbf{p}}-\textbf{p}\textbf{p}^T \end{array} \right] \end{aligned}$$

$D_{\textbf{p}}$ is a diagonal matrix with elements $\textbf{p}$, and $\nabla \theta$ is the gradient of $\theta (\Lambda ,\textbf{p},q)$ with respect to $\{q,p_1,...,p_J\}$.

Using the notation of this lemma, denote $\sigma ^2=\nabla \theta ^T\Sigma \nabla \theta$. Note that, by Eq. 1, the value of $\sigma ^2$ can be determined by the values of $\theta (\Lambda ,\textbf{p},q)$ and $\textbf{p}$ when $\Lambda$ is known. Thus, we will alternatively denote this value as $\sigma ^2(\theta ,\textbf{p})$.

#### Theorem 1

In an arbitrary hospital, let $\theta (\Lambda ,\textbf{p},q)$ denote its SIR with respect to a reference population consisting of *I* reference hospitals. Let $\hat{\theta }(\Lambda ,\hat{{\textbf {p}}},\hat{q})$ be the estimator for $\theta (\Lambda ,\textbf{p},q)$ described in Lemma 1.

Consider a hypothesis test with null hypothesis $\theta =1$ and alternate hypothesis $\theta >1$. If the null is rejected when

4 $$\begin{aligned} \frac{\hat{\theta }(\Lambda ,\hat{{\textbf {p}}},\hat{q})-1}{\sigma (1,\textbf{p})/\sqrt{n}} > z_{1-\alpha } \end{aligned}$$

The power ($1-\beta$) to detect a $\theta$ of at least $1+\delta$, while allowing for a type I error rate of $\alpha$, can be described by the following equation:

5 $$\begin{aligned} 1-\beta =\frac{1}{I} \sum _{i=1}^I \bigg [1-\Phi \bigg (\bigg (z_{1-\alpha }-\frac{\delta }{\sigma (1,\textbf{p}^{(i)})/\sqrt{n}}\bigg )\times \frac{\sigma (1,\textbf{p}^{(i)})}{\sigma (1+\delta ,\textbf{p}^{(i)})} \bigg )\bigg ] \end{aligned}$$

Where $\textbf{p}^{(i)}=\{p^{(i)}_1,...,p^{(i)}_J\}$ is the covariate distribution of the $i^{th}$ reference hospital, $\Phi$ is the cumulative density function of the standard normal distribution, and $z_{1-\alpha }$ is the value of $\Phi$ at $1-\alpha$.

Equation 5 describes a monotonic relationship between $\beta$ and *n*, allowing us, for fixed values of $\alpha , \beta ,$ and $\delta$, to easily compute *n* through a variety of existing univariate root-finding algorithms (for example, [17]).

Of note is the fact that Eq. 5 does not contain any information from the index hospital. This is especially important in the context of sample size calculation, where data for the target population is typically unavailable. One way to address this issue is to assume $\sigma ^2(\theta ,\textbf{p})$ to simply take whatever value would result in the highest required sample size to achieve the desired power. However, the value of $\textbf{p}$ which would maximize $\sigma ^2(\theta ,\textbf{p})$ may be unlikely to occur in real life, leading this approach to demand an unnecessarily high sample size. Another approach would be to simply use the overall covariate distribution of the reference population as an estimator for $\textbf{p}$. This approach, however, assumes a stable covariate distribution across hospitals. Alas, this is not the case. Covariate distributions vary substantially across hospitals, and we must account for the uncertainty accordingly. Thus, we believe, and intend to show, that Eq. 5 presents the best means of sample size calculation in the (highly likely) event that one has no information about the index hospital for which one is performing sample size calculations.

## Simulation study

We evaluated our proposed methodology by testing whether the computed required sample size can identify high SIR values. This process is engaged by simulating fictional hospitals from a basis of real-life, observed hospitals from the University of California, San Francisco International Dose Registry (hereby known as the UCSF Registry).

### Description of data

The UCSF Registry is a multi-site collaborative dataset containing nearly all (2,319,449) consecutive adult computed tomography exams from 157 hospitals performed between November 1, 2015 and Jan 30, 2018, including 850,701 abdomen exams, 607,593 chest exams, 86,654 combined abdomen-chest exams, and 774,501 head exams. Such hospitals include public, private, academic, and non-academic institutions, from a variety of localities in Europe, Japan, and throughout the United States, representing very diverse demographics and radiological practices.

At the time of the UCSF Registry being made available for use by this paper, three of its constituent hospitals were identified as incomplete or possibly erroneous. These three hospitals (totaling only 25 examinations) were removed from consideration for this paper.

To evaluate one aspect of the quality of these radiological practices, we perform indirect standardization on the hospitals, with the outcome of interest being whether an exam has high radiation dosage. This is measured by observing whether each exam has a dose value (specifically dose length product or DLP) above a value predetermined to be high for the anatomic area. These values are 1160 mGy-cm (milliGray-centimeters) for abdomen exams, 660 mGy-cm for chest exams, 1580 mGy-cm for combined abdomen-chest exams, and 1060 mGy-cm for head exams. Evaluation of this outcome is controlled for by two categorical variables, the aforementioned anatomic area scanned, as well as the “size category” of the anatomic area scanned, denoting whether the body part is very small, small, large, or very large, determined by the diameter of the body part scanned. These two categorical variables are collapsed into one for purposes of indirect standardization, the manner described at the beginning of Methodology section.

The expected prevalence of high dose within each combination of anatomic area and size category is computed by taking the observed prevalence within all exams in the UCSF Registry. This produced highly variable prevalences, with $7\%$ probability of high dose for the smallest patients undergoing combined abdomen-chest exams and $51\%$ probability of high dose for the largest patients undergoing abdomen exams. The high impact of anatomic area and patient size category on dose suggest a need to control for their distributions in hospital profiling.

The between-hospital variance of high dose prevalence is high, ranging from $0\%$ in the best-performing hospital to $75\%$ in the worst-performing hospital. The between-hospital variance does not disappear after controlling for anatomic area scanned and size category, with SIR values ranging from 0 in the best-performing hospital to 3.0 in the worst-performing hospital.

While this wide range of observed SIRs helps illustrate the benefits hospital profiling and standardization of radiological practice can provide, it does not help assess our proposed sample size calculation methodology. In the context of our hospital profiling problem, there is little clinical interest in identifying hospitals with low SIR (for example, SIR below 1.1), as their doses are low enough that they do not need help optimizing their radiological practices. There is also little reason to power a hypothesis test to detect hospitals with very high SIR (above 1.5), because while we do wish to detect hospitals of this kind, we also expect such hospitals to be very easy to detect, regardless of the statistical methodology used.

Thus, we evaluated our proposed methodology under the hypothetical scenario of comparing a null hypothesis of SIR=1 to a minimal detectable alternate hypothesis of SIR=1.2. These are the extreme values for which our selected type I and type II error rates are meant to apply, and our methods can not be viewed as successful unless error rates fall below target values even at these values of the true SIR. Neither of these two exact values, however, were observed among the true SIR values of the example hospitals. We thus simulate a new set of index hospitals so the behavior of our methods can be evaluated under these circumstances of disproportionate clinical interest.

### Description of simulation procedure

Our simulation procedure is a five-step process:

1. Hospitals in the UCSF Registry are randomly separated into two groups. The first group, consisting of 103 hospitals, will serve as the “reference population,” while the remaining 51 hospitals will serve as the basis upon which fictional index hospitals are simulated; refer to these 51 hospitals as “base index hospitals.”
2. For each base index hospital, we construct 11 “simulated index hospitals.” These 11 simulated index hospitals have a covariate distribution identical to that of their corresponding base index hospital, but with the number of high dose exams adjusted to achieve one of 11 pre-selected SIR values. These 11 SIR values are described by a sequence of numbers starting at 0.5, ending at 1.5, increasing in increments of 0.1.
3. We compute the minimal sample size required to detect an SIR of 1.2 using our proposed methodology.
4. For each simulated index hospital, we sample a number of data points equal to the minimal sample size required. We then conduct the testing necessary to compare a null hypothesis of SIR=1 to an alternate hypothesis of SIR>1.
5. For each simulated index hospital, repeat the previous step 1000 times, letting us compute the simulated hospital’s type I error rate if the null hypothesis is true, and its type II error rate if the null hypothesis is not true.

The precise means by which we construct the simulated index hospitals described in step 2 of the simulation procedure can be found in the appendix.

Specifically for SIR values 1.0 and 1.2, we also perform steps 3-5 of this simulation two more times, using more traditional models of the SIR rather than our methodology. The two more traditional approaches are:

1. Fixed Denominator from Index Sample - We assume the SIR denominator to be known after preliminarily sampling 100 data points from the index hospital, allowing for an estimate of the covariate distribution. This method was described at the end of Description of traditional indirect standardization and its short-comings section.
2. Fixed Denominator from Reference Mean - We assume the SIR denominator to be known and equal to the covariate distribution of the overall reference population. This method was described at the end of Proposed solution section.

We conclude by comparing the type I error rates and type II error rates produced by our methodology with these two more traditional approaches.

### Simulation results

According to our proposed methodology, 613 exams need to be sampled from an index hospital to detect a SIR of 1.2 with $80\%$ power.

We see in Table 1 that, using our proposed methodology, simulated hospitals with SIR value 1.2 have an average type II error rate of $20\%$, matching the target $20\%$. The average type I error rate for simulated hospitals with SIR value 1 averages $2.5\%$, lower than the target $5\%$.

**Table 1:** Average type I and type II error rate of simulated hospitals with SIR values ranging from 0.5 to 1.5, using our proposed method

True SIR	0.5	0.6	0.7	0.8	0.9	1.0
Type I Error Rate	0.0$\%$	0.0$\%$	0.0$\%$	0.0$\%$	0.003$\%$	2.5$\%$
True SIR	1.1	1.2	1.3	1.4	1.5	
Type II Error Rate	70.9$\%$	20.0$\%$	1.7$\%$	0.01$\%$	0.0$\%$	

**Fig. 1:**
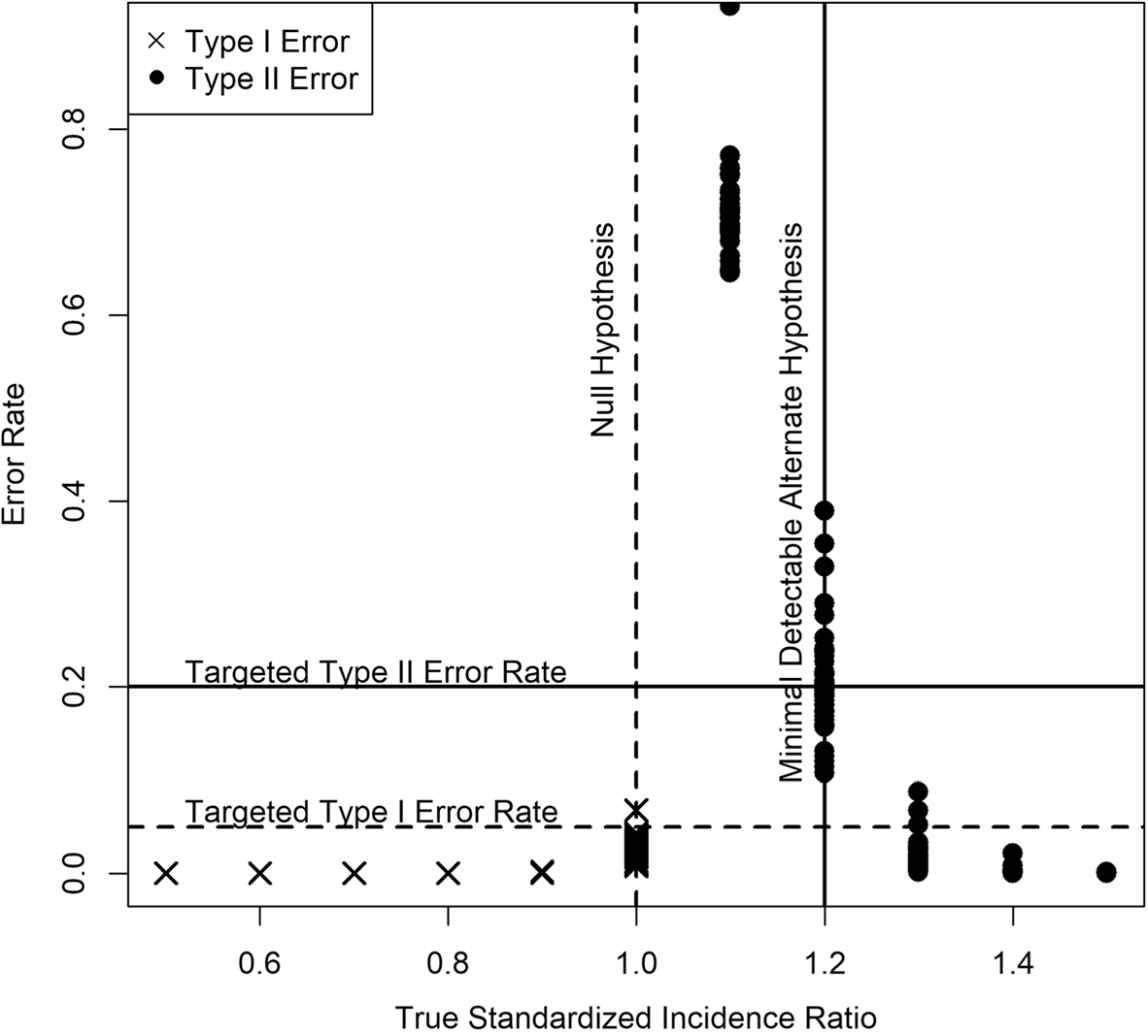
Scatter plot showing performance of our methodology for simulated hospitals with true SIR values between 0.5 and 1.5

At all other SIR values, simulated hospitals performed as expected. From Fig. 1, we see that hospitals with SIR less than 1 typically had type I error rate lower than $5\%$, while those with SIR greater than 1.2 typically had type II error rate lower than $20\%$. Hospitals with SIR of 1.1 were typically undetected, though this is expected given our choice of minimal detectable alternate hypothesis.

Lastly, we compare our methods to traditional indirect standardization, which assumes the denominator of the SIR to be known exactly.

**Fig. 2:**
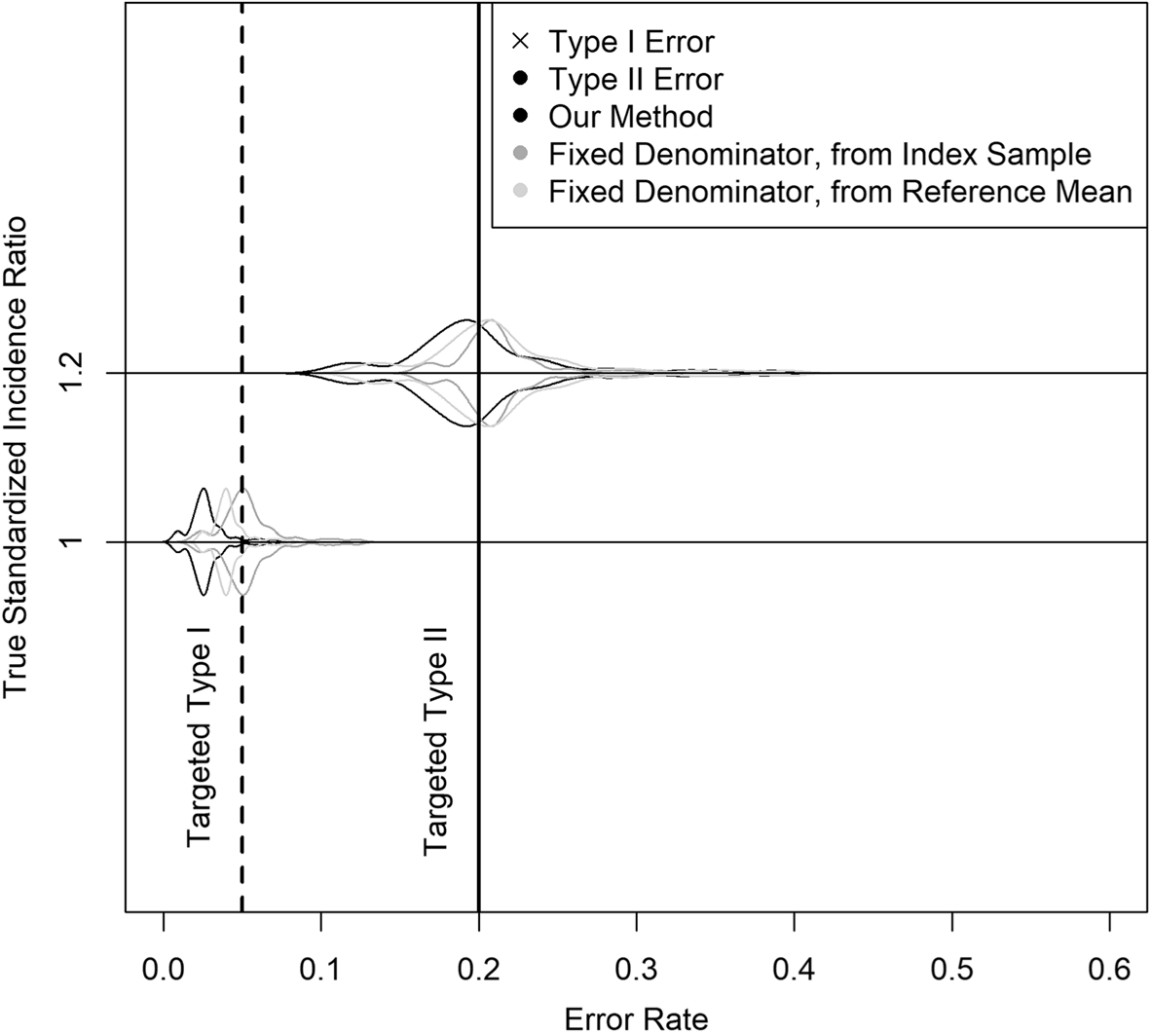
Violin plot showing performance of our methods compared to traditional methods for simulated hospitals with true SIR values 1 and 1.2

According to the methodology assuming fixed denominator from index sample, sample size required ranges from 438-989 exams, depending on the index hospital in question, with a median of 610 and an interquartile range of 75 (577-652). Among simulated index hospitals, $51\%$ required sample size below 613 according to this fixed denominator method. This results in this method often being less capable of detecting SIR values modestly higher than one. This traditional method had higher type II error rates than our methods for $90\%$ of simulated hospitals with true SIR 1.2. Viewing the SIR denominator as known also means that any inaccuracies in its estimation are also more likely carry over to errors in inference. As a consequence, even the type I error rates for traditional methods also fall below expectations, despite type I error usually decreasing when type I error to increases. Traditional methods have a higher type I error rate than our method for $73\%$ of simulated hospitals with true SIR 1 (Fig. 2).

According to the methodology assuming fixed denominator from reference mean, sample size required is 610. This traditional method also underperforms compared to our proposed method most of the time. Among simulated hospitals with true SIR of 1.2, this method had higher type II error rate than our proposed method $88\%$ of the time. Among simulated hospitals with true SIR of 1.1, this method had higher type I error rate $86\%$ of the time (Fig. 2).

## Application to real data

Lastly, to see the performance of our methodology on real data, we re-apply our methods to index hospitals drawn directly from the UCSF Registry, rather than hospitals simulated using the UCSF Registry as a base. For this exercise, we wish for the “base index hospitals” to represent a wide range of SIR values. To achieve this, we consider six categories of SIR values: <0.9, 0.9-0.95, 0.95-1, 1-1.25, 1.25-1.5, >1.5. From each category, we sample either 2 hospitals or 1/3 of all available hospitals in the category to serve as index hospitals, whichever number is higher. The resultant counts for number of index and reference hospitals in each SIR category are detailed in Table 2.

**Table 2:** Number of hospitals randomized as index and reference

	<0.9	0.9-0.95	0.95-1	1-1.25	1.25-1.5	>1.5
Index Hospitals	31	2	1	6	2	8
Reference Hospitals	62	4	3	12	6	17

Using the resulting set of reference hospitals, our methodology computes that a sample size of 615 is required to detect 1.2 SIR with $80\%$ power. Just like in simulations, for each index hospital, we sample 615 patients 1000 times to assess the type I error (for index hospitals with true SIR $\le$ 1) or type II error (for index hospitals with true SIR>1) of our methodology.

**Fig. 3:**
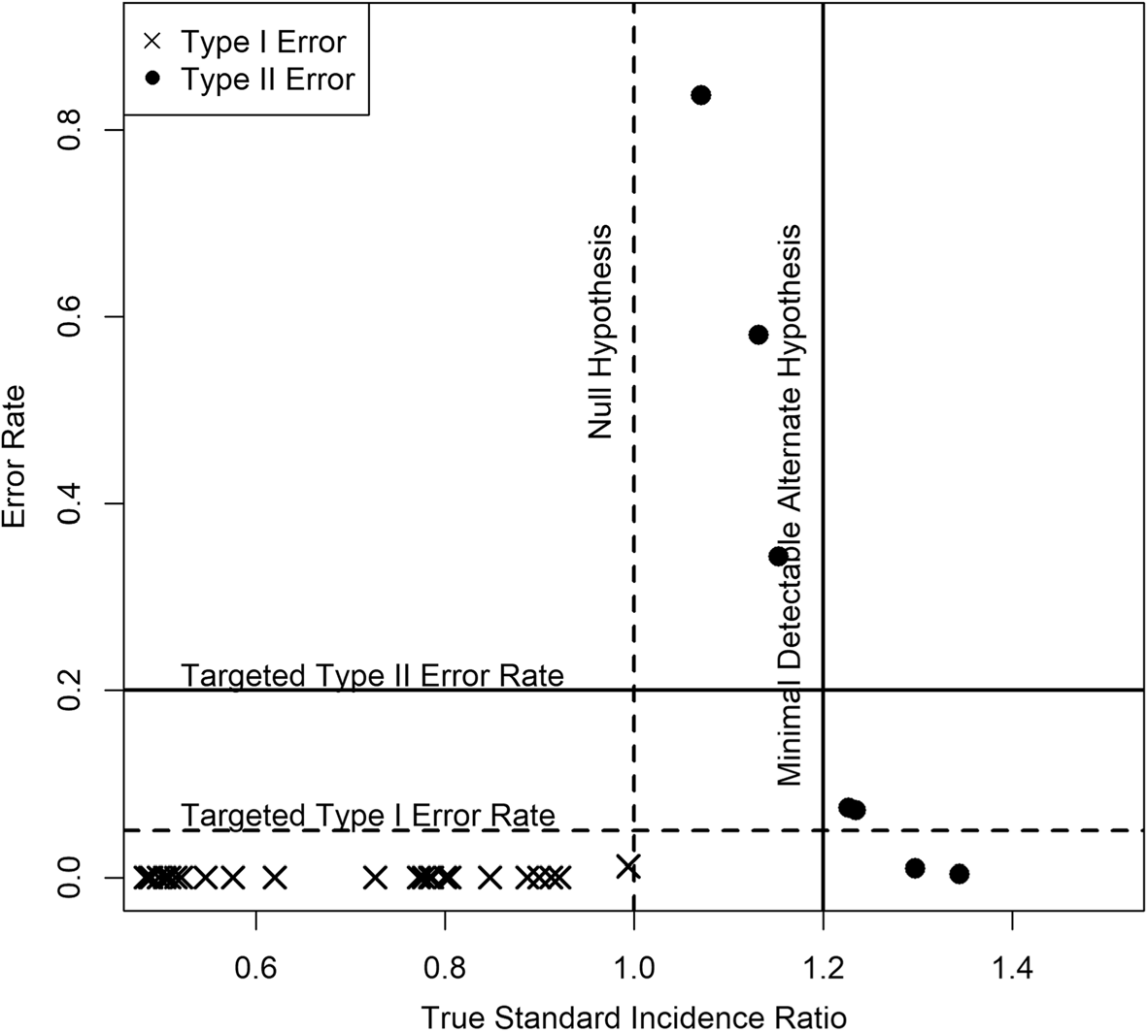
Scatter plot showing performance of our methodology for true SIR values between 0.5 and 1.5. Out of 51 index hospitals, 18 had true SIR values fall within this region. The remaining 33, for clarity, were not included in the figure, though all had very low error rates

According to Fig. 3, these expectations were met. Type I error rates fell below $5\%$ for all hospitals with true SIR less than 1. Type II error rates fell below $20\%$ for all hospitals with true SIR greater than 1.2.

## Discussion and conclusion

The ability to compare one’s own performance with the performance of other hospitals is an extremely important component of hospital profiling. To do this in a nuanced method that controls for confounding variables, however, involves one hospital sharing information with another to a degree which may not be logistically feasible or may require navigating legal and policy issues that, at best, significantly slow down the process and, at worst, render the process impossible. Thus, as much as possible, it’s of great merit to reduce the amount of information that needs to be shared, and finding the minimal sample size necessary for a proper confounder-adjusted comparison is key to addressing this problem. We provide a method of calculating this minimal sample size without requiring the same information as traditional methods. Indeed, we do not require any information from the index hospital.

When conducting simulated sample size calculations using traditional assumptions of the SIR, we were very generous in the resources theoretically considered available. Specifically, what’s described as the “fixed denominator from index sample” is highly infeasible to apply in practice, as few hospitals would be willing to engage in the circular practice of providing a sample of data to a statistical collaborator for the purposes of finding out how much data needs to be sampled. Despite this dynamic, our proposed method has been shown to work better than traditional methods.

The sample sizes required in our example application also seems modest enough to upload into a small, easy-to-use web application which can provide hospital profiling services in seconds without excessive communication between the parties being compared. Development of this web application is the ultimate goal to which this paper hopes to contribute. We hope for this web application to contribute to an expansion of hospital profiling and ultimately to an increase in quality of patient care.

While the motivating medical problem of this paper lie in the realm of optimization of radiological practices, the applications of indirect standardization are broad, extending to domains such as cardiology [7], pulmonology [8], demography [6], and many others. We expect the methods presented in this paper to be applicable in many domains outside of its original intent.

### Supplementary Information

**Additional file 1.** Sample Dataset for Application of Proposed Methodology (data.csv). To protect patient confidentiality, the hospitals providing the example data used in this paper have not given permission for the data to be made publicly available. We have, however, included a limited “fake” version of the dataset. This dataset contains 3 variables - dlp.over indicates whether an exam is “high dose,” sizeC is an ID indicating the combination of anatomic area examined and patient size category, while fac is an ID indicating the hospital the exam was performed in. Information on which ID values are associated with which anatomic areas, patient sizes, and hospital will not be provided, as they are not necessary for the illustration of statistical methods described in the paper. Note that since the dataset made available is different from the dataset used in the paper, the results should be expected to be comparable, but not identical. The software implementing the methods described in this article is available on request from the author.

## Data Availability

The dataset supporting the conclusions of this article is included within the article (and its additional files). The software implementing the methods described in this article is available on request from the author. To protect patient confidentiality, the hospitals providing the example data used in this paper have not given permission for the data to be made publicly available. We have, however, included a limited “fake” version of the dataset so readers may themselves implement the methods described in this paper. Note that since the dataset made available is different from the dataset used in the paper, the results should be expected to be comparable, but not identical.

## Appendix

### Statement of (multivariate) delta method

Proof of Lemma 1 involves use of the delta method, which we will re-state here for convenience of the reader.

#### Theorem 2

Consider a vector of values $M=\{\mu _1,\mu _2,...,\mu _K\}$, which is being estimated by $\hat{M}=\{\hat{\mu }_1,\hat{\mu }_2,...,\hat{\mu }_K\}$ using a sample of *n* data points. If

$$\sqrt{n}(\hat{M}-M)\rightarrow N(0,S)$$

where $\rightarrow$ denotes convergence in distribution, then for any continuous function $h(\cdot )$ with domain consisting of real-valued vectors of length *K* and range consisting of all real (scalar) values, we have

$$\sqrt{n}(h(\hat{M})-h(M))\rightarrow N(0,\nabla h(M)^TS\nabla h(M))$$

### Proof of Lemma 1

#### Proof

Since *q* is the probability parameter of a binomial random variable and $\textbf{p}$ is the probability parameter of a multinomial random variable, we can use the asymptotic normality of both random variables to obtain

$$\sqrt{n}(\hat{q} - q)\rightarrow N(0, q(1-q))$$

$$\sqrt{n}(\hat{{\textbf {p}}} - \textbf{p})\rightarrow N(0, D_{\textbf{p}}-\textbf{p}\textbf{p}^T)$$

where “$\rightarrow$” denotes convergence in distribution.

Since *q* and $\textbf{p}$ are viewed as independent in traditional indirect standardization, we see that $\hat{p}$ and $\hat{{\textbf {p}}}$ are jointly normal. That is,

$$\sqrt{n}(\{ \hat{q},\hat{{\textbf {p}}}\} - \{ q,\textbf{p}\} ) \rightarrow N(0, \Sigma )$$

By this equation and by Eq. 1, we apply the delta method (Theorem 2) to arrive at

$$\sqrt{n}(\hat{\theta }(\Lambda ,\hat{{\textbf {p}}},\hat{q}) -\theta (\Lambda ,\textbf{p},q)) \rightarrow N(0,\nabla \theta ^T\Sigma \nabla \theta )$$

This completes the proof. 

### Proof of Theorem 1

#### Proof

By the result of Lemma 1, we see that rejecting the null when

$$\frac{\hat{\theta }(\Lambda ,\hat{{\textbf {p}}},\hat{q})-1}{\sigma (1,\textbf{p})/\sqrt{n}} > z_{1-\alpha }$$

achieves a type I error of less than $\alpha$.

We next note that, since $\sigma ^2(\theta ,\textbf{p})$ depends on $\theta$, the left hand side of the equation above is only asymptotically normal under the null hypthesis. Under an alternative hypothesis where $\theta (\Lambda ,\textbf{p},q)=1+\delta >1$, additional computations must be done. The probability of rejecting the null under the alternate hypothesis - that is, the power - can be computed as followers

$$\begin{aligned} 1-\beta&= \Pr \bigg (\frac{\hat{\theta }-1}{\sigma (1,\textbf{p})/\sqrt{n}}> z_{1-\alpha }\bigg )\\ \Rightarrow 1-\beta&=\Pr \bigg (\frac{\hat{\theta }-(1+\delta ) }{\sigma (1+\delta ,\textbf{p})/\sqrt{n}} > \bigg (z_{1-\alpha }-\frac{\delta }{\sigma (1,\textbf{p})/\sqrt{n}}\bigg )\times \frac{\sigma (1,\textbf{p})}{\sigma (1+\delta ,\textbf{p})}\bigg )\\ \Rightarrow 1-\beta&=1-\Phi \bigg (\bigg (z_{1-\alpha }-\frac{\delta }{\sigma (1,\textbf{p})/\sqrt{n}}\bigg )\times \frac{\sigma (1,\textbf{p})}{\sigma (1+\delta ,\textbf{p})}\bigg ) \end{aligned}$$

Note that for fixed $\alpha$ and $\delta$, the value of $1-\beta$ increases as *n* increases, an expected relationship between sample size and power. Also note that, as parametrized above, $1-\beta$ can not be deterministically computed if $\textbf{p}$ is unknown. In the event that $\textbf{p}$ is unknown, we apply the law of total expectations to get

$$\begin{aligned} 1-\beta&=\oint \Pr \bigg (\frac{\hat{\theta }-1}{\sigma (1,\textbf{p})/\sqrt{n}} > z_{1-\alpha }|\textbf{p}=\textbf{p}'\bigg )f_\textbf{p}(\textbf{p}')\text {d}\textbf{p}'\\ \Rightarrow 1-\beta&=\oint \bigg ( 1-\Phi \bigg (\bigg (z_{1-\alpha }-\frac{\delta }{\sigma (1,\textbf{p}')/\sqrt{n}}\bigg )\times \frac{\sigma (1,\textbf{p}')}{\sigma (1+\delta ,\textbf{p}')}\bigg )\bigg )f_\textbf{p}(\textbf{p}')\text {d}\textbf{p}' \end{aligned}$$

where $\oint$ integrates over the support of possible values of $\textbf{p}$ and $f_{\textbf{p}}$ is the density function over this support. We next approximate this support of $\textbf{p}$ and $f_{\textbf{p}}$ using the collection of covariate distributions observed among reference hospitals. In other words, we approximate the support of $\textbf{p}$ using $\{\textbf{p}^{(1)},\textbf{p}^{(2)},...,\textbf{p}^{(I)}\}$.

$$\begin{aligned} 1-\beta&\approx \frac{1}{I}\sum _{i=1}^I \Pr \bigg (\frac{\hat{\theta }-1}{\sigma (1,\textbf{p})/\sqrt{n}} > z_{1-\alpha }|\textbf{p}=\textbf{p}^{(i)}\bigg )\\ \Rightarrow 1-\beta&\approx \frac{1}{I} \sum _{i=1}^I \bigg [1-\Phi \bigg (\bigg (z_{1-\alpha }-\frac{\delta }{\sigma (1,\textbf{p}^{(i)})/\sqrt{n}}\bigg )\times \frac{\sigma (1,\textbf{p}^{(i)})}{\sigma (1+\delta ,\textbf{p}^{(i)})} \bigg )\bigg ] \end{aligned}$$

This completes the proof.   

### Mechanism for constructing simulated hospital

For a given base index hospital and a desired SIR, we do the following

1. Compute the expected incidence of high dose exams, given covariate distribution in the base index hospital, with conditional probabilities of the outcome drawn from the reference population.
2. Compute what the incidence of high dose exams would be if the SIR were equal to the target value. This is done by multiplying the target SIR with the expected incidence of high dose exams.
3. Compute the difference of the observed incidence of high dose exams minus the incidence of high dose exams under the target SIR value.
4. If this difference is greater than 0, we randomly select that number of high dose exams from the fake index hospital and replace their outcome values with not high dose.
5. If this difference is less than 0, we randomly select that number of not high dose exams from the fake index hospital and replace their outcome values with high dose.
6. The resulting dataset is viewed as the collection of all exams performed in the simulated index hospital, from which one can then sample any number of manifestations.

## Abbreviations

SIR: standardized incidence ratio
UCSF: University of California, San Francisco
